# Vae Victis. Prophecy of Non-Panel Practitioners Fulfilled

**Published:** 1913-11-29

**Authors:** W. E. Barton

**Affiliations:** Streatham, S.W.


### VAE VICTIS.

### Prophecy of Non-Panel Practitioners Fulfilled.

*To the Editor of* The Hospital.

Sir,—It was frequently prophesied by those medical men who preferred freedom in the exercise of their profession to the bondage of panel practice under the Insurance Act, that those who decided to go on the panel would very soon experience what the sacrifice of their liberty entailed, that having placed themselves under the heel of the Insurance Commissioners they would be ground down lower and lower. More recently it has been very well said that "the panel doctors have no rights and few privileges," and on reading through the latest form of agreement which they are expected to sign it is quite evident that this is a true aphorism.

Many of these doctors who favoured the panel system frequently expressed their satisfaction in this system of wholesale contract practice, stating there would be no sending out of bills and no anxiety about payment for services rendered, but, on the contrary, there would be a nice little cheque coming in every quarter and no clerical work involved in obtaining it. But all these lovely theories have been dispelled; the amount of clerical work increases daily, statistics increase, accounts for payment have to be sent to the Commissioners every quarter, so that unless the panel doctor employs a secretary the greater part of his spare time will be taken up in complying with the Regulations as regards work done, statistics, etc., the latter being absolutely valueless from a National Health point of view. Nor is this all; he is in a position to be called to order and not paid for his services if he does not comply with all the Regulations, and be hauled up in the County Court by any cantankerous individual who is not satisfied with his medical service. He is dependent entirely for his remuneration on the number of register cards, notwithstanding that he may have only received one card out of 300, as stated by Dr. Burgess in the Supplement of the *British Medical Journal*, November 15, p. 431. Dr. Grant also exclaims in the Supplement of the same journal, November 22, p. 462: "If our salaries are to depend on the number of register cards we receive, then heaven help the doctors on the London panel." He is also bound by Regulation 2 (ii) not to accept any fee or other remuneration in respect of treatment which he is required to give, etc., etc. Consequently, if his exceptional services call forth the gratitude of any of his patients and he be asked to accept a present of a box of cigars or even a plump wurzel-fed pheasant, he would have to refuse acceptance, otherwise his conduct would be declared illegal and he would be condemned by the Insurance Commissioners and probably be reprimanded. To such a state of serfdom has the noble profession descended. —Yours faithfully,

W. E. Barton.

Streatham, S.W., November 22, 1913.

---

"Non-Panel," Kennington.—Will you be good enough to send your name and address to the Editor, The Hospital, 28 and 29 Southampton Street, Strand, W.C.?

